# HDAC6 Inhibition Protects against OGDR-Induced Golgi Fragmentation and Apoptosis

**DOI:** 10.1155/2019/6507537

**Published:** 2019-07-02

**Authors:** Jie Zhang, Jieqiong Tan, Zhiping Hu, Chunli Chen, Liuwang Zeng

**Affiliations:** ^1^Department of Neurology, Second Xiangya Hospital, Central South University, Changsha, Hunan 410011, China; ^2^Center for Medical Genetics, School of Life Sciences, Central South University, Changsha 410078, China

## Abstract

The Golgi apparatus (GA) is a pivotal organelle, and its fragmentation is an essential process in the development of apoptosis. GA is a potential target in the treatment of cerebral ischemia-reperfusion injury. Histone deacetylase 6 (HDAC6) catalyzes the removal of functional acetyl groups from proteins and plays an important role in cell homeostasis. In this study, the neuroprotective effects and the underlying mechanisms of HDAC6 inhibition were assessed in an ischemia-reperfusion injury model. Mouse neuroblastoma N2a cells and cultured neurons were subjected to oxygen-glucose deprivation/reperfusion (OGDR) insult. OGDR induces Golgi fragmentation and reduces tubulin acetylation in N2a cells and cultured neurons. Golgi fragmentation is prior to nuclear chromatin condensation after OGDR injury. Overexpression of GBF1 not only protects against OGDR-induced Golgi fragmentation but also protects against OGDR-induced apoptosis, suggesting that Golgi fragmentation is not secondary to apoptosis but plays a causal role for subsequent apoptosis. HDAC6 inhibition suppresses OGDR-induced tubulin deacetylation, p115 cleavage, and caspase 3 activation and protects against OGDR-induced Golgi fragmentation and apoptosis. This work opens a new avenue for potential clinical application of HDAC6 inhibitors for cerebral ischemia-reperfusion-related disorders.

## 1. Introduction

The Golgi apparatus (GA) is a pivotal organelle for glycosylation and membrane traffic. It plays a key role in the pathophysiological process of many disorders, and we have summarized the critical role of GA in signal transduction and cell apoptosis after cerebral ischemia-reperfusion injury and other oxidative stress-related diseases [1]. Brief histotoxic hypoxia induced vacuolizations of GA in cultured cortical and hippocampal CA1 neurons [2]. Fragmentation of GA was found in human patients with stroke, Alzheimer's disease, amyotrophic lateral sclerosis (ALS), chronic atrial fibrillation, and many other disorders [3–5]. We have also found that oxygen-glucose deprivation/reperfusion (OGDR) insult induced Golgi fragmentation [6].

Fragmentation of GA is an early apoptotic event independent of the cytoskeleton [7]. Golgi fragmentation and subsequent collapse are an essential process in the development of apoptosis. Golgi fragmentation is not secondary to apoptosis but it may “trigger” apoptosis [8]. miR-497 promotes neuronal death after cerebral ischemia by inhibiting antiapoptotic proteins bcl-2 and bcl-w [9]. PPARdelta overexpression protects against oxygen-glucose deprivation-induced cerebral vascular endothelial cell death by suppressing caspase 3 activity, Golgi fragmentation, and increasing bcl-2 protein level [10]. We have also demonstrated that Hsp20 protects against OGDR-induced Golgi fragmentation and apoptosis through the Fas/FasL pathway [6]. Therefore, GA is supposed to be a potential therapeutic option in cerebral ischemia-reperfusion injury. Golgi fragmentation is associated with unstable microtubules and inhibited acetylation of tubulin [11], while the acetylation of tubulin is regulated by two opposing enzymes, HDAC6 (deacetylation) and *α*-tubulin acetyltransferase-1 (*α*TAT1; acetylation).

HDAC6 exists exclusively in the cytoplasm and deacetylates cytoplasmic proteins such as tubulin. It plays critical roles in cell homeostasis in both physiological and pathological conditions. HDAC6 inhibitors exert neuroprotection in both cellular and animal models of ischemic stroke [12]. Tubastatin A, an HDAC6 inhibitor, attenuates cerebral infarction size and functional deficits. The neuroprotective mechanisms of Tubastatin A are related to HDAC6 inhibition and the subsequent upregulation of acetylated tubulin [13].

Therefore, on the basis of previous findings, the aims of this study are as follows: (1) to determine whether OGDR induced tubulin deacetylation in N2a cells and cultured neurons, (2) to determine the relationship between Golgi fragmentation and apoptosis after OGDR injury, and (3) to determine whether HDAC6 inhibition protected against OGDR-induced Golgi fragmentation and apoptosis. In this study, we confirmed that OGDR induced Golgi fragmentation and tubulin deacetylation in N2a cells and cultured neurons. Golgi fragmentation was prior to nuclear chromatin condensation, and overexpression of GBF1 protected against OGDR-induced Golgi fragmentation and apoptosis. HDAC6 inhibition attenuated OGDR-induced tubulin deacetylation, p115 cleavage, caspase 3 activation, Golgi fragmentation, and apoptosis. Our results suggest that GA is a promising target in the therapy of cerebral ischemia-reperfusion injury.

## 2. Materials and Methods

### 2.1. Mouse N2a Neuroblastoma Cell Culture

Mouse N2a neuroblastoma cells were purchased from the American Type Culture Collection (ATCC). N2a neuroblastoma cells were used and maintained in Dulbecco's modified Eagle's medium (DMEM), supplemented with 10% FBS (Gibco BRL), 100 U/mL penicillin, and 100 *μ*g/mL streptomycin, at 37°C in a moist atmosphere containing 5% CO_2_. To induce differentiation, growth medium was replaced with an equal volume of serum-free DMEM for 36 h.

### 2.2. Primary Neuron Culture

Cerebral cortices from mice were isolated in ice-cold EBSS. Tissue was digested with papain (Worthington Biochemical Corp., 3120) for 10 min at 37°C. The digestion was terminated using a digestion inhibition solution (EBSS containing 5 mg/mL BSA (Sigma-Aldrich, A2153), 5 mg/mL trypsin inhibitor (Sigma-Aldrich, T9253), and 10 mg/mL DNase (Sigma-Aldrich, DN25)). The tissue solution was filtered into a 50 mL tube. Collected cells were resuspended in seeding medium (DMEM containing 10% FBS) and seeded on poly-D-lysine treated plates. 6 hours later, the seeding medium was replaced with a neurobasal medium (Invitrogen, 21103049) including B-27 (Invitrogen, 0080085SA).

### 2.3. OGDR

To mimic ischemic-like conditions *in vitro*, cell cultures were exposed to oxygen-glucose deprivation (OGD) for 4 hours and then returned to 95% air, 5% CO_2_, and glucose-containing medium for a different recovery time as before [14]. First, mouse N2a neuroblastoma cells or cultured neurons were transferred into a temperature-controlled (37°C) anaerobic chamber (Forma Scientific) containing a gas mixture composed of 5% CO_2_ and 95% N_2_. The culture medium was replaced with deoxygenated glucose-free Hanks' Balanced Salt Solution (Invitrogen), and cells were maintained in the hypoxic chamber for 4 hours. After OGD, N2a cells or cultured neurons were maintained in DMEM supplemented with 10% FBS under normoxic culture conditions for 2 or 4 hours.

### 2.4. Antibodies, Plasmids, and Chemicals

The antibodies, plasmids, and chemicals used in this study include the anti-p115 antibody (ab184014, 1 : 1000; Abcam), cleaved caspase 3 (#9661, 1 : 1000, Cell Signaling Technology), HDAC6 (#7558, Cell Signaling Technology, 1 : 1000, Abcam), acetylated tubulin (T7451, 1 : 10,000 for WB, 1 : 1000 for IF, Sigma-Aldrich), tubulin (#2144, 1 : 10,000 for WB, 1 : 1000 for IF; Cell Signaling Technology), and TGN38 (T9826, 1 : 200, Sigma-Aldrich). 4′,6-Diamidino-2-phenylindole (DAPI) and tubacin were obtained from Sigma-Aldrich. For the shHDAC6 construct, an oligo was synthesized for knockdown mouse HDAC6. The sequence is: CCGGTGAGGATGACCCTAGTGTATTCTCGAGAATACACTAGGGTCATCCTCATTTTTG. The oligo and scramble oligo were ligated to the pLKo.1-GFP vector. The GBF1-GFP plasmid was obtained from AMS Biotechnology Ltd.

### 2.5. Immunofluorescence Staining

Mouse N2a neuroblastoma cells or cultured neurons grown on coverslips were subjected to OGD for 4 hours followed by reperfusion for 2 or 4 hours. The cells were then fixed with 4% paraformaldehyde for 30 min and washed three times with PBS, pH 7.4. The cells were incubated with a primary antibody overnight at 4°C. On the following day, the cells were incubated with a fluorescein-conjugated secondary antibody for 1 h. Cells were counterstained with 1 *μ*g/mL DAPI to visualize nuclear morphology. Slides were washed, wet mounted, and examined with an Olympus confocal fluorescence microscope. Fluorescence pictures were taken with identical exposure settings.

### 2.6. Transfection

N2a and primary culture neurons were transfected with Lipofectamine 3000 (Invitrogen) according to the manufacturer's instructions.

### 2.7. Western Blot Analysis

Total protein was isolated from the N2a cells using a 2XSDS sample buffer (63 mM Tris-HCl, 10% glycerol, and 2% SDS). Samples (20–40 *μ*g of protein) were electrophoresed onto a 10–15% SDS/polyacrylamide gel (SDS/PAGE) and transferred to PVDF membranes. The membranes were blocked in TBS-Tween buffer containing 20 mM Tris-HCl, 5% nonfat milk, 150 mM NaCl, and 0.05% Tween-20 (pH 7.5) for 1 hour at room temperature. Thereafter, the blot was incubated with primary antibody for 1–2 hours at room temperature. The membrane was washed 3 times with TBST at 10-minute intervals, incubated with the secondary antibody (1 : 5000; anti-rabbit or anti-mouse IgG conjugated with horseradish peroxidase; Jackson ImmunoResearch Laboratories Inc.) at room temperature for 1 hour, then washed 3 times with TBST at 10-minute intervals and 2 times with TBS, each for 10 minutes. A band was visualized via an enhanced chemiluminescence kit (ECL) according to the manufacturer's suggested protocol (GE Healthcare). Membranes were then exposed to X-ray film.

### 2.8. Measurement of Apoptosis

Mouse N2a neuroblastoma cells transfected with pLKo.1-shRNA HDAC6 and scramble shRNA were subjected to OGD for 4 hours followed by reperfusion for 4 hours. Apoptosis was detected by the Annexin V-FITC Apoptosis Detection Kit (Sigma-Aldrich). Briefly, N2a cells were collected and washed twice with PBS. 500 *μ*L binding buffer suspension was then added to the treated cells. After that, 5 *μ*L Annexin V-FITC and 10 *μ*L propidium iodide were added to each group and cultures were incubated at 37°C for 5~15 min in the dark. A flow cytometer (BD Biosciences) was used to detect the percentage of cells with apoptosis, and FlowJo software was used for flow cytometry analysis.

### 2.9. Quantitative and Statistical Analysis

For quantitation, fragmented Golgi was defined as scattered dots (not connected) in the perinuclear region or multiple mini-Golgi (isolated dots) dissociated from the major GA [5]. Quantification was performed using more than 300 cells per experiment. Quantitative analysis of selected bands in western blots was performed by using the NIH Image Analysis System (ImageJ 1.48 version). Quantitative data were expressed as mean ± SEM based on at least 3 separate experiments of triplicate samples. Differences among groups were statistically analyzed by one-way analysis of variance followed by Bonferroni's post hoc test. Comparison between two experimental groups was based on a two-tailed *t*-test. Differences between the mean values were considered significant if *P* < .05.

## 3. Results

### 3.1. OGDR Induces Golgi Fragmentation and Reduces Tubulin Acetylation in N2a Cells

**Figure 1:**
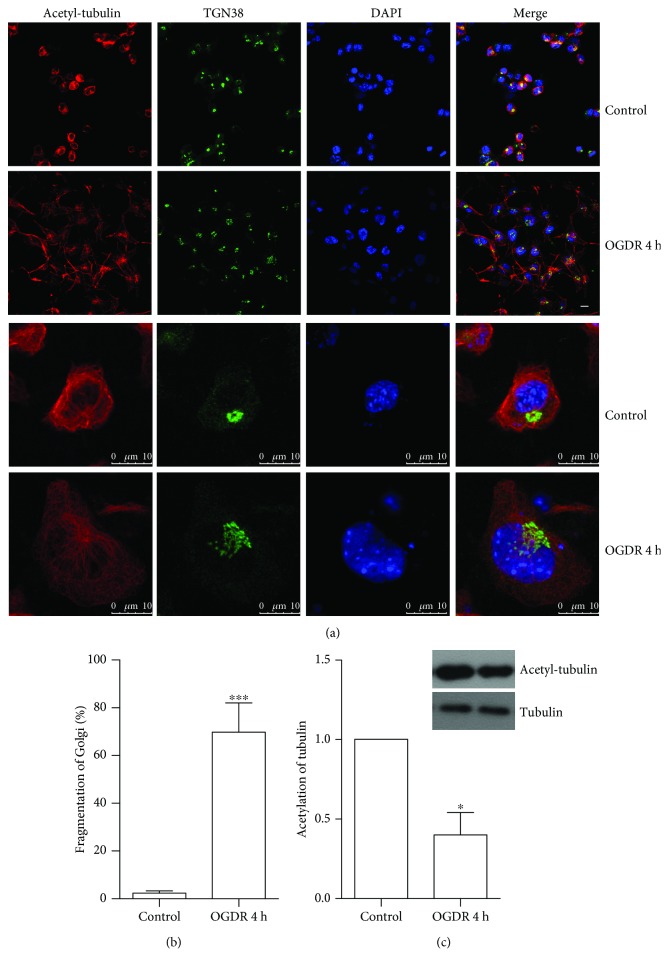
Fragmentation of GA and tubulin acetylation in N2a cells after OGDR. The experiment was repeated independently for at least three times. (a) Immunofluorescent stain using antibodies against acetylated tubulin (red color) and Golgi marker TGN38 (green color) and counterstain with 4,6-diamidino-2-phenylindole (blue color) to show nuclei revealed normal GA in normal conditions. However, more and more N2a cells showed fragmented GA after 4 h reperfusion following 4 h OGD. (b) Quantitation (mean ± SEM) of (a) from three independent experiments. The proportion of N2a cells with fragmented GA increased after 4 h reperfusion following 4 h OGD exposure. (c) Quantitation (mean ± SEM) of (a) from three independent experiments. Tubulin acetylation was significantly downregulated after 4 h reperfusion following 4 h OGD in N2a cells. The western blot data also showed that OGDR insult decreased tubulin acetylation in N2a cells. ^∗^*P* < 0.05 and ^∗∗∗^*P* < 0.001 compared to normal conditions. Bar = 10 *μ*m.

To explore whether Golgi fragmentation occurs in N2a cells after OGDR insult, we used immunofluorescent staining to evaluate its temporal profiles (Figure 1). The increase of Golgi fragmentation was found after OGDR insult. As demonstrated in Figure 1(a), most of GA appeared to be ribbon-like structures adjacent to the nuclei in normal conditions. After 4 h reperfusion following 4 h of OGD, the morphology of GA changed to debris-like structures scattered in the cytoplasm (Figures 1(a) and 1(b)).

By using immunofluorescence staining, Figure 1 depicts tubulin acetylation of N2a cells after OGDR treatment. Data from these experiments indicated that acetylated tubulin was significantly decreased after 4 h reperfusion following 4 h OGD in N2a cells (Figures 1(a) and 1(c)). Consistent with the immunofluorescence assay, the western blot data also showed that OGDR insult inhibited tubulin acetylation in N2a cells (Figure 1(c)).

### 3.2. Golgi Fragmentation and Tubulin Acetylation in Cultured Neurons after OGDR Insult

**Figure 2:**
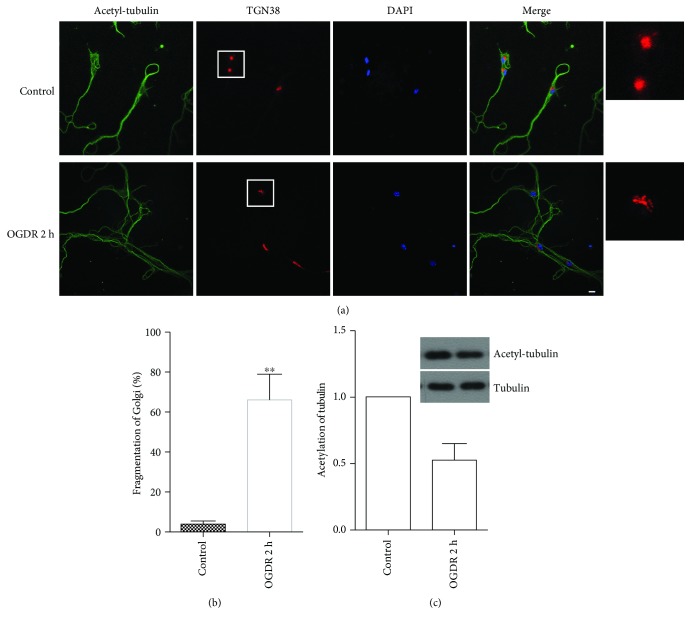
Alteration of Golgi fragmentation and tubulin acetylation in cultured neurons after OGDR. The experiment was repeated independently for at least three times. (a) Immunofluorescent stain using antibodies against acetylated tubulin (green color) and Golgi marker TGN38 (red color) and counterstain with 4,6-diamidino-2-phenylindole (blue color) to show nuclei revealed ribbon-like GA adjacent to the nuclei in the control group. However, Golgi fragmentation increased greatly in cultured neurons after 2 h reperfusion following 4 h OGD. (b) Quantitation (mean ± SEM) of (a) from three independent experiments. The proportion of cultured neurons with fragmented GA increased after 2 h reperfusion following 4 h OGD exposure. (c) Quantitation (mean ± SEM) of (a) from three independent experiments. Tubulin acetylation was significantly decreased after 2 h reperfusion following 4 h OGD in cultured neurons. The western blot data also showed that OGDR insult decreased tubulin acetylation in cultured neurons. ^∗^*P* < 0.05 and ^∗∗^*P* < 0.01 compared to normal conditions. Bar = 10 *μ*m.

We cultured neurons in vitro to further confirm that ODGR induced Golgi morphology alteration. As demonstrated in Figure 2, Golgi fragmentation was also increased in cultured neurons after OGDR insult. Most of ribbon-like GA adjacent to the nuclei changed to debris-like structures scattered in the cytoplasm after 2 h reperfusion following 4 h OGD in cultured neurons (Figure 2(b)).

We also investigated tubulin acetylation in cultured neurons. Figure 2 depicts tubulin acetylation in cultured neurons after 2 h reperfusion following 4 h OGD by using immunofluorescence staining. Data from these experiments also suggested significant reduction of acetylated tubulin in cultured neurons after OGDR injury (Figures 2(a) and 2(c)), which was consistent with the results from western blot analysis (Figure 2(c)).

### 3.3. Golgi Fragmentation Is prior to Nuclear Chromatin Condensation in N2a Cells upon OGDR Insult

**Figure 3:**
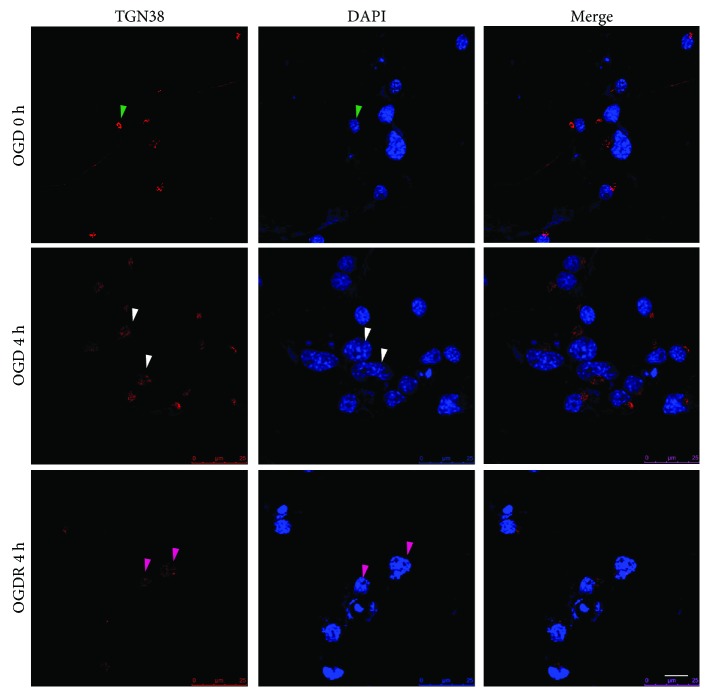
Time courses of Golgi fragmentation and nuclear chromatin condensation in N2a cells after OGDR. Immunofluorescent stain using an antibody against Golgi marker TGN38 (red color) and counterstain with 4,6-diamidino-2-phenylindole (blue color) to show nuclei. Green arrows show N2a cells with normal GA and normal nuclear chromatin without OGD insult. White arrows show N2a cells with fragmented Golgi and normal nuclear chromatin after 4 h OGD. Red arrows show N2a cells with fragmented Golgi and nuclear chromatin condensation after 4 h reperfusion following 4 h OGD. Bar = 10 *μ*m.

We further evaluated the time courses of Golgi fragmentation and nuclear chromatin condensation in N2a cells after OGDR injury by using immunofluorescent staining. As demonstrated in Figure 3, Golgi fragmentation and nuclear chromatin condensation were found after OGDR insult. However, Golgi fragmentation was obvious in N2a cells after 4 h OGD insult, while most of the cells still appeared to be normal nuclear chromatin without condensation. After 4 h reperfusion following 4 h OGD, Golgi fragmentation was exacerbated, while nuclear chromatin condensation began to appear. These results indicate that Golgi fragmentation was prior to nuclear chromatin condensation in N2a cells upon OGDR insult.

### 3.4. Overexpression of GBF1 Protects against OGDR-Induced Golgi Fragmentation and Apoptosis in N2a Cells

**Figure 4:**
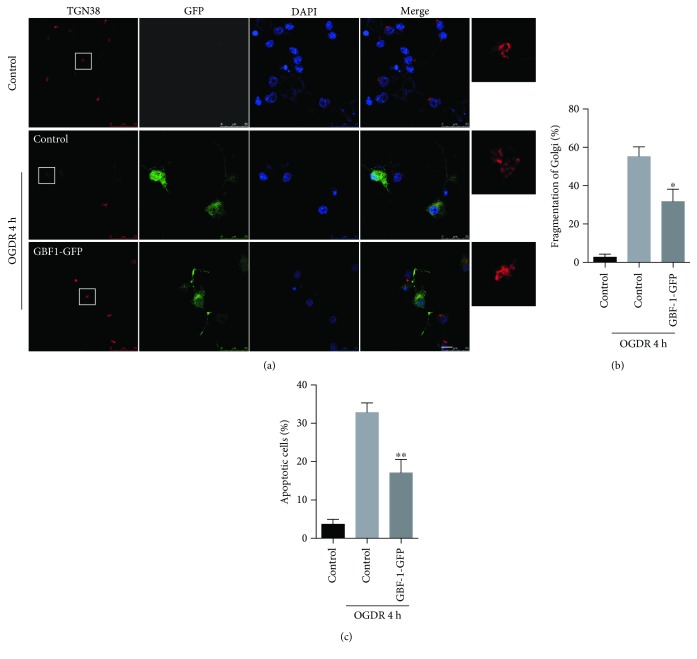
Effects of GBF1 overexpression on GA morphology and apoptosis in N2a cells exposed to OGDR. N2a cells were transfected with different plasmids (pEGFP-N1 and pEGFP-GBF1). After transfection for 36 h, cells were treated with 4 h OGD plus 4 h reperfusion. The experiment was repeated independently for at least three times. (a) Digital photomicrograph under fluorescent illumination showing the morphology of GA which was detected using TGN38 staining. GA displayed typical ribbon-like structures adjacent to the nuclei in N2a cells transfected with pEGFP-N1 without OGDR exposure. Fragmented GA was evident in N2a cells transfected with pEGFP-N1 exposed to 4 h OGD plus 4 h reperfusion insult. Transfection with pEGFP-GBF1 significantly attenuated OGDR-induced fragmentation of GA. (b) Quantitation (mean ± SEM) of (a) from three independent experiments. (c) Overexpression of GBF1 in vitro reduced OGDR-induced apoptosis. Values are expressed as mean ± SEM. ^∗^*P* < 0.05 compared to control. ^∗∗^*P* < 0.01 compared to control. Bar = 10 *μ*m.

We overexpressed GBF1 in N2a cells to assess the relationship between OGDR-induced Golgi fragmentation and apoptosis. N2a cells were transfected with different plasmids (pEGFP-N1 and pEGFP-GBF1). After transfection for 36h, cells were treated with 4 h OGD plus 4 h reperfusion. As demonstrated in Figure 4(a), GA displayed typical ribbon-like structures adjacent to the nuclei in normal N2a cells without OGDR exposure. When N2a cells transfected with pEGFP-N1 were exposed to 4 h OGD plus 4 h reperfusion, lots of GA changed to debris-like structures scattered in the cytoplasm. However, transfection with pEGFP-GBF1 significantly attenuated OGDR-induced Golgi fragmentation (Figures 4(a) and 4(b)).

To determine whether GBF1 overexpression also affected the induction/or efficiency of apoptosis, after transfection with pEGFP-N1 and pEGFP-GBF1 plasmids for 36 h, N2a cells were subjected to 4 h OGD plus 4 h reperfusion (Figure 4(c)). The percentage of apoptotic cells was increased after 4 h OGD plus 4 h reperfusion. However, N2a cells transfected with pEGFP-GBF1 displayed a significant decrease in the number of apoptotic cells after OGDR exposure. Together, these data strongly suggest that increased GBF1 expression ameliorates OGDR-induced Golgi fragmentation and apoptosis.

### 3.5. HDAC6 Inhibition Protects against OGDR-Induced Golgi Fragmentation in Cultured Neurons and N2a Cells

**Figure 5:**
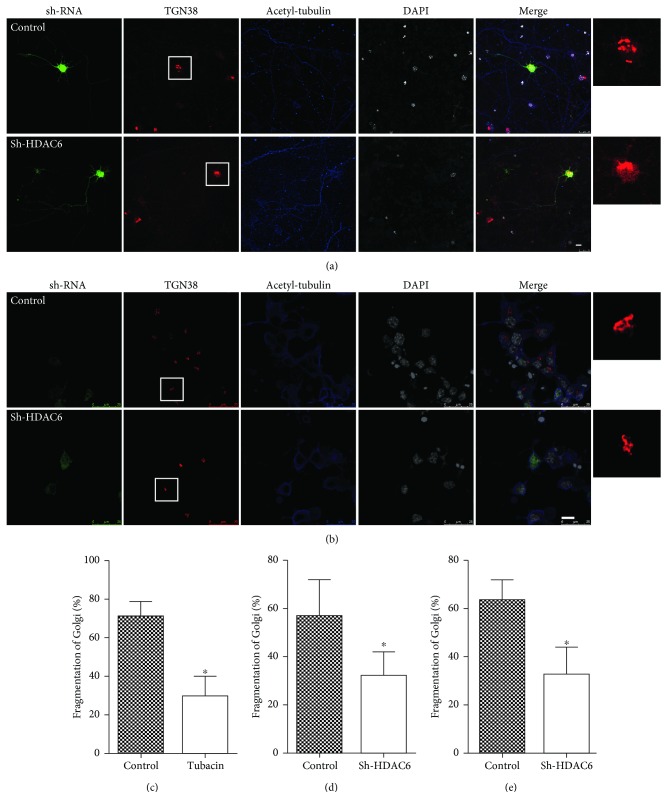
Effect of HDAC6 inhibition on GA morphology in cultured neurons and N2a cells exposed to OGDR. Cultured neurons and N2a cells were transfected with different shRNA. After transfection for 36 h, cultured neurons and N2a cells were treated with 4 h OGD plus 2 h reperfusion or 4 h reperfusion, respectively. The experiment was repeated independently for at least three times. (a) Digital photomicrograph under fluorescent illumination showing the morphology of GA which was detected using anti-TGN38 staining. Fragmented GA was evident in cultured neurons transfected with control shRNA exposed to 4 h OGD plus 2 h reperfusion. Transfection with HDAC6 shRNA significantly attenuated OGDR-induced fragmentation of GA. (b) Transfection with HDAC6 shRNA also significantly attenuated OGDR-induced fragmentation of GA in N2a cells. (c) Quantitation (mean ± SEM) of (a) from three independent experiments. (d) Quantitation (mean ± SEM) of (b) from three independent experiments. (e) Pretreatment with tubacin significantly ameliorated OGDR-induced GA fragmentation in N2a cells. Values are expressed as mean ± SEM. ^∗^*P* < 0.05 compared to control. Bar = 10 *μ*m.

To assess the role of HDAC6 in the regulation of Golgi morphology after OGDR insult, we transfected cultured neurons and N2a cells with shRNA to decrease HDAC6 expression. After transfection for 36 h, cells were treated with 4 h OGD plus 2 h reperfusion for cultured neurons or 4 h reperfusion for N2a cells. As demonstrated in Figure 5, GA changed to debris-like structures scattered in the cytoplasm in cultured neurons and N2a cells transfected with control vector. However, lots of GA still displayed typical ribbon-like structures adjacent to the nuclei in cultured neurons and N2a cells transfected with HDAC6 shRNA (Figures 5(a) and 5(b), respectively). Data from these experiments suggested that the proportion of cultured neurons and N2a cells with fragmented GA decreased greatly after HDAC6 shRNA transfection (Figures 5(c) and 5(d), respectively).

We also used the HDAC6 inhibitor tubacin to further evaluate the effect of HDAC6 inhibition in the regulation of Golgi morphology after OGDR injury. N2a cells were pretreated with tubacin and then subjected to 4 h OGD plus 4 h reperfusion. It was found that OGDR-induced Golgi fragmentation was significantly inhibited by pretreatment with tubacin (Figure 5(e)).

### 3.6. HDAC6 Inhibition Ameliorates OGDR-Induced Apoptosis in N2a Cells

**Figure 6:**
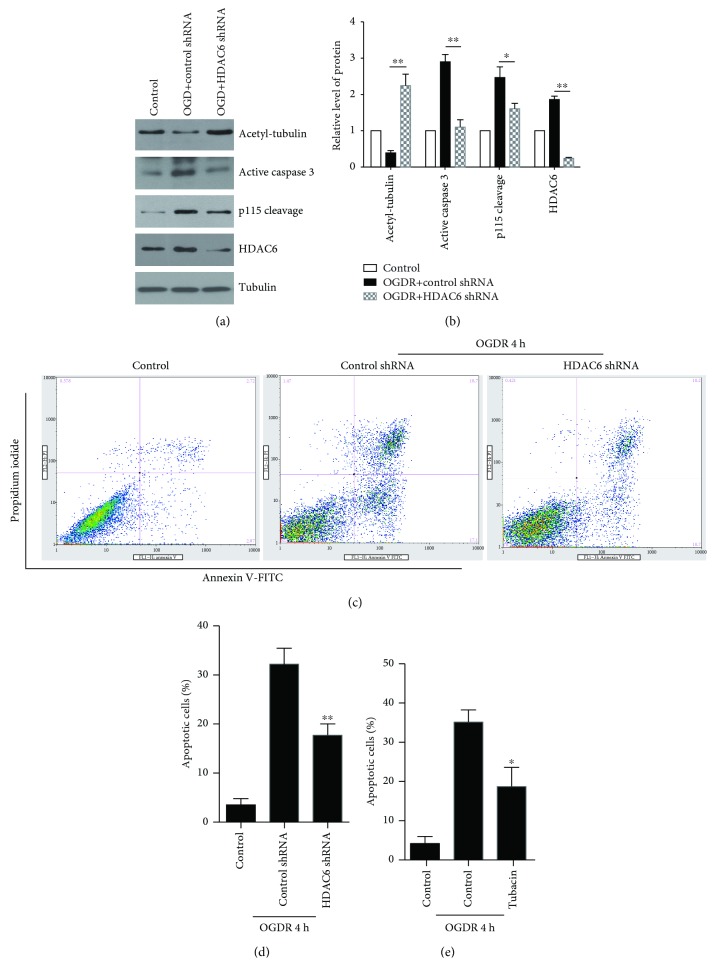
Inhibition of HDAC6 confers protection against OGDR-induced apoptosis in N2a cells. (a) Mouse N2a cells transfected with HDAC6 shRNA or control shRNA were subjected to 4 h OGD plus 4 h reperfusion, and western blot was performed to examine the protein levels of acetylated tubulin, activated caspase 3, p115 cleavage, and HDAC6. Inhibition of HDAC6 contributed to increase tubulin acetylation and decrease caspase 3 activation and p115 cleavage. (b) Quantitation (mean ± SEM) of (a) from three independent experiments. The expression of HDAC6 is downregulated after HDAC6 shRNA transfection. HDAC6 shRNA transfection also attenuated OGDR-induced tubulin deacetylation, caspase 3 activation, and p115 cleavage. (c) Downregulation of HDAC6 was able to suppress OGDR-induced apoptosis. (d) Quantitation (mean ± SEM) of (c) from three independent experiments. (e) Pretreatment with tubacin significantly ameliorated OGDR-induced apoptosis in N2a cells. ^∗^*P* < 0.05 and ^∗∗^*P* < 0.01 compared to control.

To further analyze the effect of HDAC6 inhibition on OGDR-induced apoptosis, N2a cells were transfected with control shRNA or HDAC6 shRNA. As demonstrated in Figure 6, HDAC6 shRNA transfection resulted in decreased expression of HDAC6 protein after OGDR insult. Meanwhile, our results also demonstrated that HDAC6 shRNA transfection increased tubulin acetylation and inhibited p115 cleavage and caspase 3 activation after OGDR insult (Figures 6(a) and 6(b)).

Strikingly, HDAC6 inhibition by HDAC6 shRNA transfection (Figures 6(c) and 6(d)) or pretreatment with the HDAC6 inhibitor tubacin (Figure 6(e)) suppressed OGDR-induced apoptosis. Therefore, our data proved that HDAC6 inhibition inhibited p115 cleavage and caspase 3 activation and exerted the antiapoptotic effect.

## 4. Discussion

Golgi fragmentation represents an early preclinical feature of many neurological diseases and has been widely studied in neurodegenerative disorders [4]. Golgi fragmentation is supposed to be one of the neuropathological hallmarks of ALS and other neurodegenerative diseases. We did a lot of work on the pathological alterations of GA after cerebral ischemia. Consistent with our previous results [6], in this study, we confirmed that OGDR induced Golgi fragmentation in N2a cells, as well as in cultured neurons. As we know, GA plays essential roles in the secretory pathway, engaging in control of the processing of various cellular components and membrane trafficking. Therefore, Golgi fragmentation and disruption of Golgi function after OGDR insult might cause the breakdown of overall cellular architecture and ultimately cell death. Our results indicate that protection of GA might be a promising therapeutic strategy to improve functional recovery after cerebral ischemia-reperfusion injury.

In our study, we also found that OGDR insult reduced tubulin acetylation in N2a cells and cultured neurons. The integrity of GA requires the microtubule network. TBCE gene encodes the *cis*-Golgi-localized tubulin-binding cofactor E, one of five chaperones that assist in tubulin folding and microtubule polymerization. Loss of TBCE leads to a defective tubulin polymerization and causes Golgi fragmentation [4, 15]. Microtubules enriched in acetylated tubulin are supposed to play an important role in establishing and maintaining the Golgi organization. Knockdown of KIF7 expression caused fragmentation of the Golgi network, which is a result of abnormal tubulin acetylation and microtubular dynamics [16]. Meanwhile, depletion of centrosome- and Golgi-localized protein kinase N-associated protein (CG-NAP) impedes tubulin acetylation and causes Golgi fragmentation [17]. Thus, reduced tubulin acetylation after OGDR insult may have a detrimental effect on GA integrity and contribute to Golgi fragmentation.

We further studied the relationship between Golgi fragmentation and apoptosis after OGDR injury. We found that OGDR insult not only induced Golgi fragmentation but also induced nuclear chromatin condensation in N2a cells. However, Golgi fragmentation was prior to nuclear chromatin condensation. These results indicate that Golgi fragmentation is not secondary to apoptosis but may be the initial trigger of apoptosis. GBF1 (Golgi brefeldin A-resistant factor 1), a guanine nucleotide exchange factor for Arf-GTPases, plays a central role in maintaining the structural integrity of GA. It is involved in the regulation of Golgi fragmentation [18]. GBF1 inhibition led to disassembly of the Golgi and *trans*-Golgi network [19]. We demonstrated that overexpression of GBF1 not only decreased OGDR-induced Golgi fragmentation but also ameliorated OGDR-induced apoptosis. Taken together, these results suggest that Golgi fragmentation is not secondary to apoptosis but plays a causal role for subsequent apoptosis after OGDR insult.

HDAC6 decreases the acetylation level of histones and other nonhistone proteins and regulates the ubiquitin proteasome system, autophagy, cell migration, and other cellular processes [20]. HDAC6 inhibitors are promising candidates for the treatment of neurological diseases, neoplasms, infectious diseases, and other diseases associated with HDAC6 activity [21]. In this study, we found that HDAC6 inhibition ameliorated OGDR-induced Golgi fragmentation in cultured neurons and N2a cells, suggesting that downregulation of HDAC6 exerts a neuroprotective effect in cerebral ischemia-reperfusion injury.

The neuroprotective mechanisms underlying HDAC6 inhibition are yet to be clarified. HDAC6 is a therapeutic target in the Charcot-Marie-Tooth disease [22]. HDAC6 inhibition restores tubulin acetylation, rescues disrupted axonal transport, and improves motor functions in the Charcot-Marie-Tooth neuropathy [23]. HDAC6 is involved in the necroptosis of neurons during ischemia-reperfusion by modulating the levels of ROS and acetylated tubulin [24]. We found that HDAC6 regulated tubulin acetylation after OGDR injury, and the decreased expression of acetylated tubulin was ameliorated by HDAC6 inhibition. As suggested above, OGDR-induced Golgi fragmentation may result from the reduced tubulin acetylation. Therefore, HDAC6 inhibition protects GA after OGDR insult through upregulating tubulin acetylation.

p115, the Golgi-vesicle-tethering protein, is present in the intermediate compartment and *cis*-Golgi vesicles. It is a key molecule contributing to the biogenesis and maintenance of the Golgi structure [25]. p115 associates with tubulin, and both the N- and C-terminal regions of p115 are required to maintain the Golgi structure [26]. Depletion of p115 leads to extensive Golgi fragmentation and impaired secretory traffic [27]. We found that HDAC6 inhibition ameliorated OGDR-induced p115 cleavage, which may contribute to protect the GA from fragmentation.

Histone deacetylase inhibitors are found to induce apoptosis in different tumor cell types. HDAC6 deacetylase activity inhibition by selective inhibitors inhibits glioblastoma cell proliferation and induces apoptosis [28]. WT161, a bioavailable HDAC6 inhibitor, activates caspase and augments apoptosis in multiple myeloma [29]. As for neurons, silencing of HDAC6 induces apoptosis in gonadotropin-releasing hormone neuronal cells [30]. In response to the hypoxia-ischemia after acute spinal cord injury, HDAC6 inhibition also accelerates cell apoptosis [31]. However, HPOB, an HDAC6 inhibitor, reduces corticosterone-induced apoptosis in PC12 cells and thus exerts a neuroprotective effect [32]. In our study, we found that the downregulation of the HDAC6 deacetylase activity inhibited caspase 3 activation and attenuated OGDR-induced apoptosis, suggesting the antiapoptotic and neuroprotective effect of HDAC6 inhibition after cerebral ischemia-reperfusion injury.

In combination with these research results, attenuated p115 cleavage is supposed to be involved in the antiapoptotic role of HDAC6 inhibition after OGDR insult. p115 degradation plays a key role in amplifying the apoptotic response [33]. p115 is cleaved at Asp757 by caspase 3 and generates a C-terminal fragment (CTF) of 205 residues. Endogenous CTF is translocated to the cell nucleus subsequently and promotes the apoptotic response [34]. The caspase cleavage fragment of p115 enhances apoptosis through a p53-dependent pathway [35]. Therefore, HDAC6 inhibition ameliorated OGDR-induced p115 cleavage, which may contribute to the antiapoptotic effect of HDAC6 inhibition after OGDR injury.

In conclusion, our study demonstrates that OGDR induces Golgi fragmentation and reduces tubulin acetylation in N2a cells and cultured neurons. Golgi fragmentation is prior to nuclear chromatin condensation. Overexpression of GBF1 not only protects against OGDR-induced Golgi fragmentation but also protects against OGDR-induced apoptosis, suggesting that Golgi fragmentation is not secondary to apoptosis but plays a causal role for subsequent apoptosis. HDAC6 inhibition suppresses OGDR-induced tubulin deacetylation, p115 cleavage, and caspase 3 activation and protects against OGDR-induced Golgi fragmentation and apoptosis. Our results suggest that downregulation of HDAC6 may be a promising strategy for the treatment of OGDR injury. However, further efforts are still needed to develop novel HDAC6 inhibitors for disorders whose etiology is based upon cerebral ischemia-reperfusion injury.

## Data Availability

No data were used to support this study.
